# Previous cervical cytology and high-risk human papillomavirus testing in a cohort of patients with invasive cervical carcinoma in Shandong Province, China

**DOI:** 10.1371/journal.pone.0180618

**Published:** 2017-06-29

**Authors:** Liran Zhang, Fengxiang Xie, Xinguo Wang, Dezhi Peng, Chunrui Bi, Lingbo Jiang, Dongman Zhao, Xinxin Tian, Debo Qi

**Affiliations:** 1 Department of Pathology, Jinan KingMed Diagnostics, Jinan, Shandong Province, China; 2 Department of Laboratory Medicine, Jinan KingMed Diagnostics, Jinan, Shandong Province, China; Rudjer Boskovic Institute, CROATIA

## Abstract

**Background:**

Currently, available data regarding previous cervical cytology and high-risk human papillomavirus (hrHPV) test results to detect invasive cervical cancer are limited and controversial in China. Therefore, this retrospective study in a population of Chinese women with invasive cervical carcinoma aimed to gain further insight into the roles of cytology and hrHPV testing in cervical cancer screening.

**Methods:**

A total of 1214 cases with a histological diagnosis of invasive cervical cancer were retrieved from the Pathology Database of Jinan KingMed Diagnostics (JKD) over a 5-year period. Previous cytology and hrHPV test results of 469 patients carried out within the year before cancer diagnosis were documented.

**Results:**

A higher percentage of patients who had undergone prior screening had micro-invasive cervical carcinoma than patients who had no prior screening (25.4% vs. 12.1%, P < 0.001). Of the 469 patients with available prior screening results, 170 had cytology alone, 161 had hrHPV testing alone, and 138 had both cytology and hrHPV testing. There was a significantly lower percentage of hrHPV-positive cases with adenocarcinoma than with squamous cell carcinoma (77.8% vs. 96.4%, P = 0.001). The hrHPV test showed a significantly higher sensitivity than cytology alone (94.4% vs. 85.3%, P = 0.006). The overall sensitivity of the combination of cytology and hrHPV testing (98.6%) was much higher than that of cytology alone (P < 0.001) but only marginally higher than that of hrHPV testing alone (P = 0.058).

**Conclusions:**

The results revealed that prior cervical screening can detect a significantly larger number of micro-invasive cervical cancers. The hrHPV test can provide a more sensitive and efficient strategy than cytology alone. As the addition of cytology to hrHPV testing can only marginally increase the efficiency of the hrHPV test, hrHPV testing should be used as the primary screening approach, especially in the low-resource settings of China.

## Introduction

China carries a heavy burden of cervical cancer, with 12% of new cases and 11% of deaths worldwide in 2012 [1]. The cervical cancer incidence was 9.8 and the mortality was 2.6 per 100,000 Chinese women in 2010, with an overall increasing trend in both incidence and mortality in the period from 2003 to 2010 [1]. Significant disparities also exist in cervical cancer burden across different regions. In rural areas of China, such as Xiangyuan County and Yangcheng County in Shanxi Province, the cervical cancer rates are very high (up to 81 per 100,000 women) [2]. Although there are several population-based cervical cancer screening programs in China, screening is still not widely accepted. Thus, the majority of women of the appropriate age, especially in rural areas, do not have access to regular screening. A cytology-based cervical cancer prevention program is often not a viable choice due to the lack of infrastructure, qualified cytopathologists, and quality assurance systems.

The major cause of invasive cervical cancer is persistent infection with high-risk human papillomavirus (hrHPV) [3–5]; therefore, hrHPV testing can be used as a new primary screening tool. Several long-term, prospective controlled trials on women from industrialized countries followed up for at least two rounds of cervical screening confirmed a significantly lower cancer incidence after prior hrHPV testing compared with prior cytology-based screening [6–8]. This reduced cancer incidence is related to an increased lead time, which will enable extended screening intervals, require fewer cytologic smears, and potentially lower the costs of the initial screening.

Worldwide, the data for prior cytology and hrHPV test results for patients with invasive cervical cancer are limited. None of the aforementioned prospective controlled studies [6–8] in developed countries, where advanced cervical cancers are extremely rare among well-screened women, was powered to show the relative efficacy of hrHPV-based versus cytology-based screening. Moreover, the present data of prior cytology and hrHPV testing for patients with invasive cervical cancer are controversial compared with the results derived from its precursor lesions. Several retrospective studies in the US and China have reported that cytology-based screening has similar or even higher sensitivity than hrHPV testing [9–12]. Therefore, we retrospectively documented cases with a histological diagnosis of invasive cervical carcinoma from the Pathology Database of Jinan KingMed Diagnostics (JKD) over a 5-year period from January 2012 to December 2016. Examination of this population can offer more insight into understanding the advantages and limitations of cytology, hrHPV testing, and their combination for the detection of invasive cervical carcinoma.

## Patients and methods

### Patient cohorts

JKD is the largest independent pathology laboratory in Shandong Province, China. This study was approved by the institutional review committee of Jinan KingMed Diagnostics. The review committee waived the need of patients’ consent due to anonymous analyses of the data. The handling and publication of patients’ data in this study were strictly in accordance with the Declaration of Helsinki including confidentiality and anonymity. A retrospective study was initiated by a computer-based search of the pathology database. Patients with a histological diagnosis of invasive cervical cancer were identified over a 5-year period from January 2012 to December 2016. Prior cytology and hrHPV test results within the year before the cancer diagnosis were collected. Other clinical information, including age and clinical history available in the pathology database, was also recorded. All diagnoses of cervical cancer in this study were established by histopathological examinations, including cervical biopsy, endocervical curettage, diagnostic excisional procedures by loop electrosurgical excision procedures or cold knife cervical conization, and hysterectomy. The diagnoses were rendered by five histopathologists who report approximately 50,000 surgical cases per year. For those patients who had two or more follow-up results, the more/most severe lesion was documented. All slides were reviewed by three surgical pathologists (Xinguo Wang, Fengxiang Xie, and Debo Qi) to confirm the diagnosis and histological classification.

Cytology, hrHPV and surgical cervical cancer specimens were collected from a total of 227 hospitals across Shandong Province, and the cases were referred to JKD. Most cases originated from local community hospitals serving populations primarily in suburban and rural areas. Clinicians request cytology and/or hrHPV testing for a variety of reasons, mostly on the basis of routine screening or suspicious clinical findings. Cytology, hrHPV testing, and their combination were all included as test modalities. Cytology was performed at the Pathology Department and hrHPV testing was performed at the Genetics Department of JKD. Patients who had cytology or hrHPV testing reported by other hospitals or laboratories were not included in this study.

### Cytology

The three types of liquid-based cytology (LBC) preparations used were ThinPrep (Hologic, Bedford, MA, USA), Xinshiji (Shijiazhuang Xinshiji Bio-Engineering Limited Co., China), and Lituo (Lituo Biotechnology Co., China). All LBC tests in this study were performed in the Cytology Laboratory at JKD, according to the manufacturers’ instructions. The conventional Pap smears (CPS) were collected and fixed by individual clinicians and sent to the laboratory for slide preparation and interpretation. The cytology collection methods were largely decided by clinicians and patients. All cytology tests were reported using The Bethesda System 2001 terminology. The interpretations were categorized as unsatisfactory for evaluation, negative for an intraepithelial lesion or malignancy (NILM); atypical squamous cells of undetermined significance (ASC-US); low-grade squamous intraepithelial lesion (LSIL); atypical squamous cells, cannot exclude high-grade squamous intraepithelial lesion (ASC-H); high-grade squamous intraepithelial lesion (HSIL); atypical glandular cells (AGC); and cervical cancer cells. Interpretations of the cytology results were performed by a group of approximately 15 cytopathologists who report >300,000 cervical cytology tests per year. Most of them were trained by 1–1.5 years of cytology courses in the KingMed Cytology School after graduation from medical school. Details of the KingMed Cytology Training Program have been included in recent publications [13, 14].

### hrHPV testing

HPV testing was performed at the JKD Genetics Laboratory by one of two HPV testing methods. Some of the HPV tests were performed with the Hybrid Capture 2 (HC2) assay (Qiagen, Hinden, Germany), which tests for high- and intermediate-risk HPV types (16, 18, 31, 33, 35, 39, 45, 51, 52, 56, 58, 59, and 68). The other HPV tests were performed with a HPV genotyping kit (PCR-reverse dot blot hybridization method; Yaneng Bioscience Co., Shenzhen, China). Based on this method, 23 HPV types (6, 11, 16, 18, 31, 33, 35, 39, 42, 43, 45, 51, 52, 53, 56, 58, 59, 66, 68, 73, 81, 82, and 83) can be detected [15]. All of the HPV tests were carried out according to the manufacturers’ instructions.

### Statistical analysis

Data were analyzed by using statistical software (Statistical Product and Service Solutions, SPSS; version 19.0, IBM Co., USA). Differences in categorical data were assessed by the chi-squared test, and differences in continuous data were assessed by one-way analysis of variance followed by the Bonferroni test. *P* < 0.05 was considered to be statistically significant.

## Results

### Study patients

The findings leading to invasive cervical carcinoma diagnoses included a wide spectrum of abnormal cytology results, positive hrHPV testing results, and suspicious clinical findings. As shown in Table 1, a total of 1214 cases of invasive cervical cancer were diagnosed histologically during the 5-year study period at the Pathology Department of JKD. Of these, 469 cases (38.6%) had prior cytology and/or hrHPV testing within the year before the cancer diagnosis, including 408 cases of squamous cell carcinoma (SCC, 87.0%), 51 cases of adenocarcinoma (ADC, 10.9%), and 10 cases of adenosquamous carcinoma (ADSQ, 2.1%) (Table 1). Among the screened patients, we retrieved prior cytology-based screening data from 170 patients, prior hrHPV test results from 161 patients, and both cytology and hrHPV test results from 138 patients.

**Table 1:** Cervical cancer histological subtypes in the current study.

Histological Diagnosis	With prior cytology and/or HPV testing		Without prior screening results	
	No. (%)	Age, Average (Range), y	No. (%)	Age, Average (Range), y
SCC	408 (87.0)	47.9 (25–88)	658 (88.3)	48.5 (28–87)
ADC	51 (10.9)	49.0 (35–76)	68 (9.1)	49.4 (29–75)
ADSQ	10 (2.1)	47.7 (39–59)	15 (2.0)	50.7 (39–69)
Small cell carcinoma	0	—	3 (0.4)	48 (39–59)
Carcinosarcoma	0	—	1 (0.1)	49.0
Total	469 (100)	48.1 (25–88)	745 (100)	48.6 (28–87)

ADC, adenocarcinoma; ADSQ, adenosquamous carcinoma; HPV, human papillomavirus; SCC, squamous cell carcinoma.

The other 745 cases (61.4%) who underwent surgical procedures triggered by suspicious clinical findings alone, e.g., either a visible cervical lesion or vaginal bleeding, were also enrolled. These included 658 cases of SCC (88.3%), 68 cases of ADC (9.1%), 15 cases of ADSQ (2.0%), 3 cases of small cell carcinoma (0.4%), and 1 case of carcinosarcoma (0.1%).

Among the patients with or without prior screening results, 119 (25.4%) and 78 (10.5%) were diagnosed as having micro-invasive cervical cancer, respectively, indicating that prior screening detected more invasive cervical cancers in the early stage (P < 0.001). The incidence percentage of micro-invasive cervical cancer was similar among the different patient groups: those who had prior cytology alone (23.5%, 40/170), those who had prior hrHPV testing alone (24.8%, 40/161), and those who had both cytology and hrHPV testing (28.3%, 39/138). The average ages and the percentages of the different histological cancer types were similar among the SCC, ADC, and ADSQ patients with and without prior screening results.

### Results of prior cytology alone

Of the 469 patients included in this study, 170 patients had cytology alone (36.2%). The average patient age was 47.9 years old (range, 26–85 years old). The average interval between cytology and a primary cervical cancer diagnosis was 37.8 days (range, 0–329 days). Of these 170 patients, 163 women (95.9%) had cytology-based screening within 90 days before the histological diagnosis. As shown in Table 2, four types of cytological preparations were used: CPS (6 cases, 3.5%), ThinPrep (24 cases, 14.1%), Lituo (72 cases, 42.4%), and Xinshiji (68 cases, 40%). The NILM results were 0% for the CPS test, 4.2% for the ThinPrep test, 22.2% for the Lituo test, and 5.9% for the Xinshiji test.

**Table 2:** Results of prior cytology alone according to the cytological preparation.

Interpretation of the cytology-based screening	CPS	LBC			Total
		ThinPrep	Lituo	Xinshiji	
NILM	0	1 (4.2)	16 (22.2)	4 (5.9)	21 (12.4)
ASC-US	1 (16.7)	3 (12.5)	7 (9.7)	2 (2.9)	13 (7.6)
ASC-H	4 (66.7)	0	17 (23.6)	6 (8.8)	27 (15.9)
LSIL	0	0	0	1 (1.5)	1 (0.6)
HSIL	1 (16.7)	6 (25)	20 (27.8)	39 (57.4)	66 (38.8)
Ca	0	9 (37.5)	7 (9.7)	14 (20.6)	30 (17.6)
AGC	0	2 (8.3)	4 (5.6)	2 (2.9)	8 (4.7)
Unsatisfactory	0	3 (12.5)	1 (1.4)	0	4 (2.4)
Total	6 (100)	24 (100)	72 (100)	68 (100)	170 (100)

AGC, atypical glandular cells; ASC-H, atypical squamous cells, cannot exclude high-grade squamous intraepithelial lesion; ASC-US, atypical squamous cells of undetermined significance; Ca, carcinoma; CPS, conventional Pap smear; HSIL, high-grade squamous intraepithelial lesion; LBC, liquid-based cytology; LSIL, low-grade squamous intraepithelial lesion; NILM, negative for intraepithelial lesion or malignancy.

As shown in Table 3, HSIL was the most common abnormal cytology result, reported in 38.8% of the patients. Additionally, 17.6% of the patients tested had a cytology report of carcinoma. Other abnormal results such as ASC-US, ASC-H, AGC, and LSIL accounted for another 28.8%. Based on the cytology-based screening results overall, 85.3% (145/170) of all patients had abnormal findings. The percentage of abnormal cytology results for the SCC patients (86.7%, 130/150) was obviously higher than that for the ADC patients (73.7%, 14/19); however, the difference was not statistically significant (P = 0.133).

**Table 3:** Results of prior cytology alone performed within the year prior to the histological diagnosis of invasive cervical cancer.

Interpretation of the cytology-based screening	SCC (%)	ADC (%)	ADSQ (%)	Total
NILM	17 (11.3)	4 (21.1)	0	21 (12.4)
ASC-US	12 (8.0)	1 (5.3)	0	13 (7.6)
ASC-H	27 (18.0)	0	0	27 (15.9)
LSIL	1 (0.7)	0	0	1 (0.6)
HSIL	64 (42.7)	1 (5.3)	1 (100)	66 (38.8)
Ca	25 (16.7)	5 (26.3)	0	30 (17.6)
AGC	1 (0.7)	7 (36.8)	0	8 (4.7)
Unsatisfactory	3 (2.0)	1 (5.3)	0	4 (2.4)
Total	150 (100)	19 (100)	1 (100)	170 (100)

ADC, adenocarcinoma; ADSQ, adenosquamous carcinoma; AGC, atypical glandular cells; ASC-H, atypical squamous cells, cannot exclude high-grade squamous intraepithelial lesion; ASC-US, atypical squamous cells of undetermined significance; Ca, carcinoma; HSIL, high-grade squamous intraepithelial lesion; LSIL, low-grade squamous intraepithelial lesion; NILM, negative for intraepithelial lesion or malignancy; SCC, squamous cell carcinoma.

### Results of prior hrHPV testing alone

Of the 469 patients included in this study, 161 underwent HPV testing alone (34.3%). The average patient age was 47.7 years old (range, 28–78 years old). Of the patients who had only HPV testing, 72 (44.7%) had HC2 hrHPV testing and 89 (55.3%) had genotyping within the year before the histological diagnosis of cervical cancer. Additionally, 71 (98.6%) of the 72 patients with HC2 testing and 88 (98.9%) of the 89 patients with genotyping had undergone HPV testing within 90 days before the histological diagnosis. The average intervals between the two HPV tests and a primary diagnosis of cervical cancer were similar, 6.8 days (range, 0–157 days) for HC2 testing and 6.5 days (range, 0–158 days) for HPV genotyping, respectively.

The prior hrHPV test results are shown in Table 4. Of the patients with HC2 testing and genotyping, the hrHPV-positive rates of the SCC, ADC, and ADSQ patients were similar between the two HPV testing methods and were 95.5% vs. 97.3%, 80% vs. 76.9%, and 100% vs. 100%, respectively. Compared with the SCC patients, the ADC patients showed a significantly lower hrHPV-positive rate (77.8% vs. 96.4%, P = 0.001). The hrHPV-positive rate of the ADSQ patients was also higher than that of the ADC patients (100% vs. 77.8%), but the difference was not statistically significant (P = 0.297). This result was most likely due to the small sample size.

**Table 4:** Results of prior HPV testing alone within the year prior to the histological diagnosis of invasive cervical cancer.

Histo- logical Diagnosis	HPV testing				Total (n = 161)	
	HC2 testing (n = 72)		Genotyping (n = 89)			
	Negative (%)	Positive (%)	Negative (%)	Positive (%)	Negative (%)	Positive (%)
SCC	3 (4.5)	63 (95.5)	2 (2.7)	71 (97.3)	5 (3.6)	134 (96.4)
ADC	1 (20)	4 (80)	3 (23.1)	10 (76.9)	4 (22.2)	14 (77.8)
ADSQ	0	1 (100)	0	3 (100)	0	4 (100)
Total	4 (5.6)	68 (94.4)	5 (5.6)	84 (94.4)	9 (5.6)	152 (94.4)

ADC, adenocarcinoma; ADSQ, adenosquamous carcinoma; HC2, Hybrid Capture 2; hrHPV, high-risk human papillomavirus; HPV, human papillomavirus; SCC, squamous cell carcinoma.

### Prior results for the combination of cytology and hrHPV testing

A total of 138 patients had a combination of cytology and hrHPV testing within the year prior to their histological diagnosis, including 119, 14, and 5 cases of SCC, ADC, and ADSQ, respectively. The average patient age was 47.6 years old (range, 28–78 years). Of the 138 patients who underwent both cytology and hrHPV testing, 134 women (97.1%) had cytology-based screening within 90 days of their histological diagnosis. The average interval between cytology and a primary diagnosis of cervical cancer was 14.9 days (range, 0–320 days). We also included four types of cytology tests: CPS (3 cases, 2.2%), ThinPrep (32 cases, 23.2%), Lituo (35 cases, 25.4%), and Xinshiji (68 cases, 49.3%).

Of the patients for whom both hrHPV and cytology-based screening results were available, HC2 hrHPV testing and HPV genotyping data were available for 33 (23.9%) and 105 (76.1%) patients, respectively; these tests had been carried out within the year prior to the histological diagnosis of cervical cancer. Furthermore, 32/33 patients (97.0%) and 101/105 patients (96.2%) had undergone HPV testing within 90 days prior to the diagnosis. The average intervals between the two HPV testing methods and the cervical cancer diagnosis were 10 days (range, 0–187 days) and 15.5 days (range, 0–320 days) for HC2 testing and HPV genotyping, respectively.

As shown in Table 5, the positive rates of the combined testing in SCC, ADC, and ADSQ patients were 100% (119/119), 92.9% (13/14), and 80% (4/5), respectively. Of the patients for whom both tests were done, 115 (83.3%) had abnormal cytology/positive hrHPV test results, 2 (1.4%) had negative cytology (NILM)/negative hrHPV test results, 8 (5.8%) had abnormal cytology/negative hrHPV test results, and 13 (9.4%) had negative cytology (NILM)/positive hrHPV test results. When examining the results of the cytology in these patients within the context of the hrHPV test results, the abnormal cytology rate was 89.8% (115/128 patients) among hrHPV-positive patients. These findings indicated that a hrHPV-positive test result and abnormal cytology were correlated with each other.

**Table 5:** Results of the combination of cytology and hrHPV testing within the year prior to the histological diagnosis of invasive cervical cancer.

Interpreta-tion of the cytology- based screening	HPV testing				Total (n = 138)	
	HC2 testing (n = 33)		Genotyping (n = 105)			
	Negative (%)	Positive (%)	Negative (%)	Positive (%)	Negative (%)	Positive (%)
SCC (n = 119)						
NILM	0	3 (11.1)	0	9 (9.8)	0	12 (10.1)
ASC-US	0	0	3 (3.3)	10 (10.9)	3 (2.5)	10 (8.4)
ASC-H	0	6 (22.2)	1 (1.1)	15 (16.3)	1 (0.8)	21 (17.6)
LSIL	0	0	0	1 (1.1)	0	1 (0.8)
HSIL	0	13 (48.1)	0	41 (44.6)	0	54 (45.4)
Ca	0	5 (18.5)	1 (1.1)	10 (10.9)	1 (0.8)	15 (12.6)
AGC	0	0	0	1 (1.1)	0	1 (0.8)
Total	0	27 (100)	5 (5.4)	87 (94.6)	5 (4.2)	114 (95.8)
ADC (n = 14)						
NILM	0	1 (20)	1 (11.1)	0	1 (7.1)	1 (7.1)
ASC-US	1 (20)	2 (40)	0	0	1 (7.1)	2 (14.3)
ASC-H	0	1 (20)	0	3 (33.3)	0	4 (28.6)
HSIL	0	0	0	1 (11.1)	0	1 (7.1)
Ca	0	0	1 (11.1)	1 (11.1)	1 (7.1)	1 (7.1)
AGC	0	0	1 (11.1)	1 (11.1)	1 (7.1)	1 (7.1)
Total	1 (20)	4 (80)	3 (33.3)	6 (66.7)	4 (28.6)	10 (71.4)
ADSQ (n = 5)						
NILM	0	0	1 (25)	0	1 (20)	0
ASC-H		1 (100)	0	1 (25)	0	2 (40)
HSIL	0	0	0	2(50)	0	2 (40)
Total	0	1 (100)	1 (25)	3 (75)	1 (20)	4 (80)
All cancer cases (n = 138)						
NILM	0	4 (12.1)	2 (1.9)	9 (8.6)	2 (1.4)	13 (9.4)
ASC-US	1 (3.0)	2 (6.1)	3 (2.9)	10 (9.5)	4 (2.9)	12 (8.7)
ASC-H	0	8 (24.2)	1 (1.0)	19 (18.1)	1 (0.7)	27 (19.6)
LSIL	0	0	0	1 (1.0)	0	1 (0.7)
HSIL	0	13 (39.4)	0	44 (41.9)	0	57 (41.3)
Ca	0	5 (15.2)	2 (1.9)	11 (10.5)	2 (1.4)	16 (11.6)
AGC	0	0	1 (1.0)	2 (1.9)	1 (0.7)	2 (1.4)
Total	1 (3.0)	32 (97.0)	9 (8.6)	96 (91.4)	10 (7.2)	128 (92.8)

ADC, adenocarcinoma; ADSQ, adenosquamous carcinoma; AGC, atypical glandular cells; ASC-H, atypical squamous cells, cannot exclude high-grade squamous intraepithelial lesion; ASC-US, atypical squamous cells of undetermined significance; Ca, carcinoma; HC2, Hybrid Capture 2; HPV, human papillomavirus; HSIL, high-grade squamous intraepithelial lesion; LSIL, low-grade squamous intraepithelial lesion; NILM, negative for intraepithelial lesion or malignancy; SCC, squamous cell carcinoma.

### Comparison of the different testing methods

The sensitivities of cytology alone, hrHPV testing alone, and their combination are shown in Table 6. The overall sensitivity of hrHPV testing alone was much higher than that of cytology alone (94.4% vs. 85.3%, P = 0.006). In contrast with cytology alone, hrHPV testing alone had a higher sensitivity in SCC patients (96.4% vs. 86.7%, P = 0.003). The sensitivities of cytology alone and hrHPV testing alone, however, were similar in ADC and ADSQ patients, without a statistical difference, respectively.

**Table 6:** Comparison of the results of cytology, hrHPV testing, and their combination within the year prior to the histological diagnosis of invasive cervical cancer.

Histo- logical Diagnosis	Cytology alone (n = 170)		hrHPV testing alone (n = 161)		Combination of cytology and hrHPV testing (n = 138)	
	Sensitivity, %	95% CI	Sensitivity, %	95% CI	Sensitivity, %	95% CI
SCC	86.7 (130/150)	81.2–92.1	96.4 (134/139)	93.3–99.5	100 (119/119)	100–100
ADC	73.7 (14/19)	53.9–93.5	77.8 (14/18)	58.6–97.0	92.9 (13/14)	79.4–100
ADSQ	100 (1/1)	100–100	100 (4/4)	100–100	80 (4/5)	44.9–100
Total	85.3 (145/170)	80.0–90.6	94.4 (152/161)	90.9–98.0	98.6 (136/138)	96.6–100

ADC, adenocarcinoma; ADSQ, adenosquamous carcinoma; hrHPV, high-risk human papillomavirus; SCC, squamous cell carcinoma.

The overall sensitivity of the combination of cytology and hrHPV testing was much higher than that of cytology alone (98.6% vs. 85.3%, P < 0.001), but only marginally higher than that of hrHPV testing alone (98.6% vs. 94.4%, P = 0.058). In contrast with cytology alone, the combination of testing methods had a significantly higher sensitivity for SCC patients (100% vs. 86.7%, P < 0.001). The sensitivity of the combination of testing methods for SCC patients was also higher than that of hrHPV testing alone (100% vs. 96.4%, P = 0.037). Compared with cytology alone and hrHPV testing alone, their combination, however, had an obviously increased sensitivity for ADC patients. But the difference was not statistically significant, probably due to the small sample size.

## Discussion

In this retrospective study, the efficacies of cervical screening with cytology alone, hrHPV testing alone, and their combination were compared in a cohort of patients with invasive cervical carcinoma in Shandong Province, China. The results revealed that hrHPV testing alone had a significantly higher sensitivity than cytology (94.4% vs. 85.3%). The overall sensitivity of the combination of cytology and hrHPV testing (98.6%) was only marginally higher than that of hrHPV testing alone. These results are consistent with those from previous randomized, controlled trials, which exhibited that hrHPV testing is more sensitive than cytology for identifying cervical cancer and its precursors in population screening [16–19], indicating the applicability of a new hrHPV-based primary cervical cancer screening strategy in the rural and suburban areas of China.

The results of the current study showed that prior screening can detect more patients with micro-invasive cervical carcinoma (25.4%) than patients who do not undergo any prior screening (12.1%). In well-organized developed countries, the main task of cervical cancer screening is to find precancerous lesions; thus, invasive cervical cancers especially advanced cervical cancers, are extremely rare among women who have been screened. In Magee-Women’s Hospital in the US, only 287 cases of cervical SCC were rendered over a 7-year period between January 2004 and July 2011 [12]. Unfortunately, in China, the largest developing country, many women never have even one screening, especially in rural areas. A large pooled analysis in China has suggested that the cervical cancer incidence in China may be underestimated because of the absence of a well-established nationwide cancer registry [2]. During a short study period of 46 months, a total of 3714 cases of invasive cervical cancer were diagnosed at the Obstetrics and Gynecology Hospital of Fudan University (OGHFU), one of the largest women’s hospitals in China [9]. Among approximately 50,000 surgical cases per year in our pathology department, 1214 cases of cervical carcinoma were documented during a 5-year period. The abovementioned results indicate that China has a high incidence of cervical cancer; therefore, the timely detection of invasive cancers, especially in their early stage, is one of the main tasks for cervical cancer screening in China.

In the present study, the overall cytology-negative rate was 12.4% for the patients who had cytology-based screening alone within 1 year of a histological diagnosis of cancer. The majority (95.9%) of these patients were screened within 3 months, with an average time of only 37.8 days. This finding indicates that cytology performed even in the presence of an invasive cancer has a significant negative rate. Our current findings are similar to the results from OGHFU, where the false-negative rate was 15.5% for all cytology tests [9]. However, a much lower cytology-negative rate (1.9%) has been reported by Guangzhou KingMed Diagnostics (GKD) [10], which is the largest independent pathology laboratory fully certified by the College of American Pathologists (CAP) in China. This finding might be attributed to the high-level cytology services at GKD, with the laboratory workload standards and quality control practices all performed consistently with current CAP Laboratory Accreditation Program checklists. The different performances of cytology among these Chinese cytology laboratories indicate that implementation of strict quality control practices is fundamental to maintain a high sensitivity for interpretation of the invasive cervical cancer screening results.

This retrospective study revealed a hrHPV-positive rate of 94.4% when the test was conducted within the year prior to histological diagnosis (98.8% of the cases were tested within 90 days), and this rate was much higher than that obtained with the cytology-based screening test (85.3%). In a recent study by OGHFU in China, similar negative rates have been reported for both cytology-based screening (15.5%) and hrHPV testing (15.5%) [9]. However, studies by GKD in China [10] and three other studies in the US [11, 12, 20] have reported lower prior negative rates for cytology than for hrHPV testing. As mentioned previously, this difference might be attributed to the high-level cytology services at GKD and cytology laboratories in the US.

Currently, many challenges for cervical cancer screening still exist in China. In resource-poor regions, such as rural areas, a cervical cancer prevention program based only on cytology is not often viable because of the lack of the necessary infrastructure, qualified cytopathologists, a national cervical cancer screening program, and a national standard for cytology quality control. The additional sensitivity of hrHPV testing and the potential for high-throughput automated testing make it an attractive alternative to cytology for primary screening. Moreover, application of hrHPV testing in primary screening can also reduce the dependence of qualified cytopathologists. However, there is concern about the efficacy of hrHPV testing in cervical cancer screening in China, given that recent studies [21, 22] have shown that the hrHPV-positive rates in Chinese women were markedly higher than those reported in other populations with established cervical screening programs [8, 23, 24]. The prevalence of hrHPV has been found to correlate well with cervical cancer risk and the incidence of cervical cancer [25]. In cross-sectional, population-based studies, which mainly have been performed in rural areas of China, a high prevalence of both hrHPV and cervical intraepithelial neoplasia (CIN) 3 or worse lesions have been reported [26, 27]. These studies have confirmed that hrHPV testing is more sensitive than cytology and visual inspection for the detection of CIN 3 or worse in unscreened Chinese women. Our present results also indicate that hrHPV testing is considerably more sensitive than cytology in invasive cervical cancer screening. These results are consistent with those from well-screened populations [8, 23, 24], indicating that hrHPV testing also can be used as a primary screening tool in China. Although hrHPV testing is not recommended as a primary screening test in young women in developed countries [28, 29], the high sensitivity and specificity of hrHPV testing in young Chinese women suggest that a different strategy might be applicable in China [26].

Using a combination of cytology and hrHPV testing has been shown previously to not only detect significantly more CIN 2/3 or worse but is also associated with significantly lower rates of invasive cancer and its precursor lesions in subsequent screening rounds [8, 17, 18, 23, 30]; however, the screening cost is substantially increased by doubling the number of tests and only marginally improving the sensitivity compared with hrHPV testing alone [8, 17, 18, 23, 30]. The combination of hrHPV testing with cytology-based screening has been shown to lead to a considerably lower positive predictive value [11, 17, 28, 31, 32], thus increasing the number of positive screening results by more than a third compared with hrHPV testing alone and consequently increasing the number of women referred to colposcopy [17, 24, 27]. In addition, a large study from Kaiser Permanente Northern California showed that normal cytology caused only a slight further reduction in the already low cancer risk for hrHPV-negative women (3.2 vs. 3.8 per 100,000 women per year) [23]. Compared with hrHPV testing alone, the present study also showed that using a combination of hrHPV testing and cytology-based screening only slightly increased the sensitivity of the hrHPV test. On the other hand, the increased sensitivity obtained by adding cytology-based screening to hrHPV testing might be limited due to the low efficiency of cytology-based screening in China.

One drawback of HPV testing is that it is more expensive and time-consuming than cytology-based screening, and it requires sophisticated laboratory infrastructure. Although we did not compare the cost-effectiveness of cytology and hrHPV testing in this study, several studies in China and other low- and middle-income countries have already confirmed that HPV testing is more cost-effective than cytology for cervical cancer screening [33, 34]. In addition, the careHPV test (Qiagen), a simple and self-obtained HPV test that can provide results within 3 h, has already been evaluated in rural China, and its accuracy is similar to that of the HC2 test, indicating that the careHPV test is a promising primary screening method in low-resource settings [35].

Although the data presented herein can offer further insight into understanding the advantages and limitations of cytology, hrHPV testing, and their combination in detecting invasive cervical carcinoma, there are several limitations that cannot be ignored. First, JKD serves a large and diverse population from more than 900 local community hospitals, women’s health centers, clinics, and physical examination centers, where providers receive no special training or any special qualifications. However, we believe that our results are representative of the large-scale experience that realistically happens in routine cervical cancer screening practice in China. Some studies that have been performed in high-quality laboratories, such as in Beijing [16, 26, 27] and Guangzhou [10, 13], may not be generalizable to all laboratories in China. Second, JKD is a large, independent laboratory, and some patients have cytology and/or hrHPV testing performed in other hospitals and laboratories; therefore, these results were not obtainable. Third, most samples were taken in close temporal proximity to the histological diagnosis of cervical cancer, and for some, though not many, cytology and/or hrHPV testing were consequently done because these women were already symptomatic and clinically suspicious of cervical cancer, as shown in two other Chinese studies [9, 10]. This situation is different from that of a routine screening population, which is composed of mostly asymptomatic women. This bias may make both screening tests (cytology and hrHPV testing) appear more sensitive than in routine screening. Finally, because of the small sample size of ADC and ADSQ patients in this study, the statistical significance of the current results was largely limited.

## Conclusions

Cytology-based screening of cervical cancer can be used to detect a significantly large number of micro-invasive cervical cancers. On the other hand, hrHPV testing is more objective and sensitive than cytology alone for cervical cancer detection. The combination of cervical cytology and hrHPV testing can further marginally increase the efficiency of cervical screening; however, this strategy is not very cost-effective, and the increased sensitivity obtained by using a combination of cytology and hrHPV testing might be limited due to the low efficiency of cytology in China. Thus, the combination of cytology and hrHPV testing is not recommended in low-resource settings. In conclusion, the results of this study indicate that hrHPV testing may be an excellent primary screening tool for cervical cancer, especially in the low-resource settings of China.
